# Diversity of Grammars and Their Diverging Evolutionary and Processing Paths: Evidence From Functional MRI Study of Serbian

**DOI:** 10.3389/fpsyg.2018.00278

**Published:** 2018-03-06

**Authors:** Ljiljana Progovac, Natalia Rakhlin, William Angell, Ryan Liddane, Lingfei Tang, Noa Ofen

**Affiliations:** ^1^Linguistics Program, Wayne State University, Detroit, MI, United States; ^2^Department of English, Wayne State University, Detroit, MI, United States; ^3^Communication Sciences and Disorders, Wayne State University, Detroit, MI, United States; ^4^Lifespan Cognitive Neuroscience Program, Institute of Gerontology, Wayne State University, Detroit, MI, United States; ^5^Department of Psychology, Wayne State University, Detroit, MI, United States

**Keywords:** evolution of syntax, transitivity, syntactic processing, functional MRI, broca's–basal ganglia network

## Abstract

We address the puzzle of “unity in diversity” in human languages by advocating the (minimal) common denominator for the diverse expressions of transitivity across human languages, consistent with the view that early in language evolution there was a modest beginning for syntax and that this beginning provided the foundation for the further elaboration of syntactic complexity. This study reports the results of a functional MRI experiment investigating differential patterns of brain activation during processing of sentences with minimal versus fuller syntactic structures. These structural layers have been postulated to represent different stages in the evolution of syntax, potentially engaging different brain networks. We focused on the Serbian “middles,” analyzed as lacking the transitivity (vP) layer, contrasted with matched transitives, containing the transitivity layer. Our main hypothesis was that transitives will produce more activation in the syntactic (Broca's–Basal Ganglia) brain network, in comparison to more rudimentary middles. The participants (*n* = 14) were healthy adults (Mean age = 33.36; *SD* = 12.23), native speakers of Serbo-Croatian. The task consisted of reading a series of sentences (middles and transitives; *n* = 64) presented in blocks of 8, while being engaged in a detection of repetition task. We found that the processing of transitives, compared to middles, was associated with an increase in activation in the basal ganglia bilaterally. Although we did not find an effect in Broca's area, transitives, compared to middles, evoked greater activation in the precentral gyrus (BA 6), proposed to be part of the “Broca's complex.” Our results add to the previous findings that Broca's area is not the sole center for syntactic processing, but rather is part of a larger circuit that involves subcortical structures. We discuss our results in the context of the recent findings concerning the gene-brain-language pathway involving mutations in *FOXP2* that likely contributed to the enhancement of the frontal-striatal brain network, facilitating human capacity for complex syntax.

## Introduction

One of the most challenging puzzles about human languages is how to account for the immense diversity in their form and the equal ease with which all languages are learned by children, including those that are simultaneously acquiring two languages with very different grammatical properties (Aboh and Ansaldo, this volume). In this paper, we shed new light on this puzzle of “unity in diversity” by examining transitivity, i.e., a clause-structure phenomenon controlling how grammatical relations encode distinct participants in an event denoted by the verb. We report the results of an fMRI study investigating differential patterns of brain activation during processing of well-formed sentences with minimal versus fuller clause structural complexity. We look at this phenomenon from a language evolution perspective, conceptualizing sentences with different degrees of syntactic complexity as reflecting distinct stages in the evolution of language.

Relying on accepted postulates of theoretical syntax, Progovac (2015a, 2016a) proposed a gradual emergence of syntactic layers in language evolution, starting from the minimally complex intransitive small clause, gradually adding others, to accommodate transitivity, and verb finiteness. This precise reconstruction is achieved by peeling off the syntactic layers in (1), widely accepted (Adger, 2003; Carnie, 2013) to constitute the basic skeleton of the modern sentence^1^:

(1) VP/SC < vP < TP < CP

The lowest layer is the small clause (SC/VP), which accommodates a verb with one noun phrase (NP). The SC/VP is subsumed by the “little v” phrase (vP), an additional layer into which the traditional verb phrase is split (syntactic argumentation for this split is presented in the references above). This layer supports transitivity (i.e., addition of another argument, permitting a syntactic differentiation between agents and patients). The phrase that dominates the vP is Tense Phrase (TP), which accommodates the expression of tense and finiteness. TP can be dominated by the Complementizer Phrase (CP), which accommodates sentence embedding and *wh*-movement.

This approach offers a probe for investigating the sources of unity and diversity of human languages. Our argument is that the unity comes from the common (SC) core, which to a large extent determines further elaboration of structure, including its binary and its hierarchical nature. The diversity, on the other hand, comes from the options regarding which of the layers are utilized in which languages and in which constructions. This, for example, allows for a different sub-set of projections to be built in different languages, such as AspP instead of a TP. It also allows for coexistence of structures of varying degrees of syntactic complexity within a language, as per the analysis of Serbian transitive sentences (with a vP), and the corresponding middles (without a vP).

We seek support for this approach by using functional neuroimaging. In particular, we propose to capitalize on the coexistence of proto-syntactic “fossils” (i.e., constructions consisting of fewer and more basic layers of syntactic structure) alongside more modern structures (i.e., those operating with a fuller set of syntactic layers). We hypothesize that processing of structures with fewer syntactic layers relies on more ancient and more diffused neural networks, while highly layered structures require more recently evolved, densely interconnected and more specialized neural networks for quick and automatic processing (see also Ansaldo et al., 2015). We report the results of an fMRI experiment contrasting between the processing of constructions of differing structural complexity against each other in a reading recognition paradigm. We argue that the approach explored here brings together theories of syntax with theories of evolution, gives rise to specific testable neuroscientific hypotheses, and sheds novel light on the unity and diversity of human languages.

## Scientific premises and hypotheses

### Language variation: is there a common denominator?

The “unity in diversity” that underlies children's adaptability to various linguistic ecologies/environments is certainly of evolutionary significance, and we propose that the nature and trajectory of language evolution provides a key to understanding both the diversity and equal learnability of human languages. As previously noted, “language cannot be fully understood without reference to its evolution, *whether proven or hypothesized*” (Greenberg, 1966; Givón, 2002)^2^. The position that we advance here concurs with this statement: while we acknowledge that there are no definitive answers regarding how language evolved, there are specific hypotheses that can be used as tools to probe this question.

Our study focuses on transitivity: its emergence in language evolution, its typological variation in today's languages, and its representation in the brain. There is substantial diversity in the syntactic realization of transitivity across different languages. The conceptualization of sentence structure as described above provides a common denominator—the minimal intransitive clause—for the appearance of the various types of transitivity attested across languages: not only the nominative-accusative type (characterized by subjects receiving distinct case-marking irrespective of the presence of additional arguments, and in opposition to objects), but also ergative-absolutive type [in which agents of transitive verbs are marked distinctly (ergative case) from objects and single arguments of intransitive verbs (absolutive case)]. It also accounts for serial verb patterns, where two intransitive clauses, rather than just one, provide a foundation for constructing a single transitive sentence.

**Figure 1:**
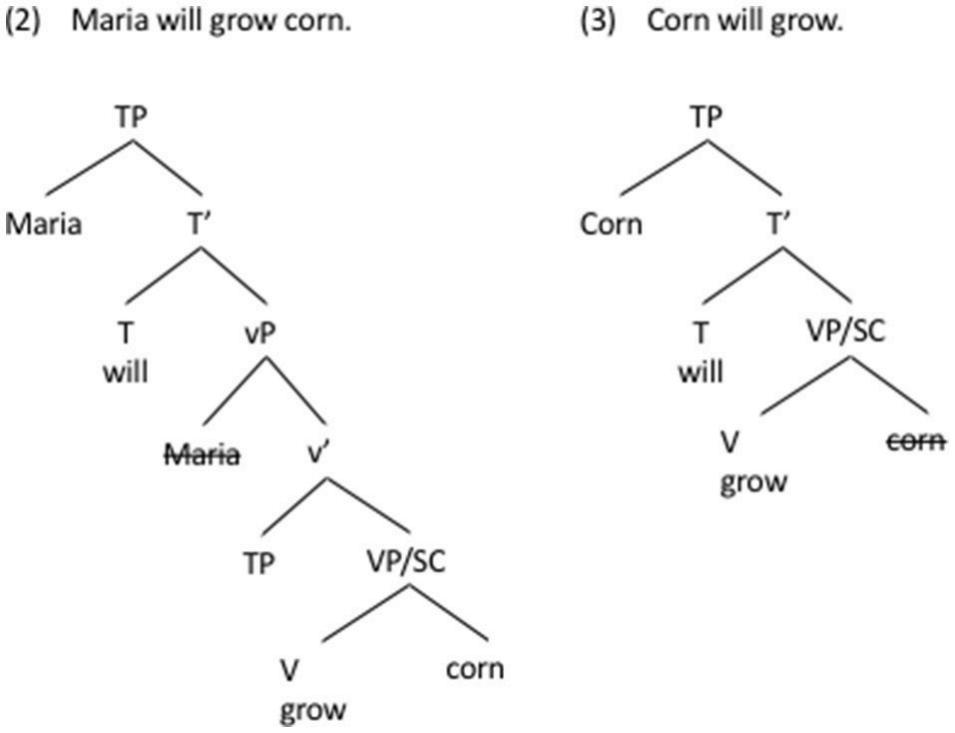
We present two sentences from English of varying degrees of syntactic elaboration. (2) illustrates a typical transitive TP (Tense Phrase), where each argument is generated in its own layer: the agent (Maria) in the vP layer, and the patient (corn) in the VP/SC layer. (3) is a hierarchically less complex, intransitive sentence, which lacks the agent and the vP layer.

As discussed above, Progovac's (2015a; 2016a) reconstruction of the gradual emergence of syntactic layers in language evolution is based on certain well-established theoretical postulates of syntactic theory (e.g., Chomsky, 1995; Adger, 2003; Carnie, 2013), which include the (partial) hierarchy of syntactic layers for the modern sentence, as introduced in section 1^3^:

In Figure 1 we present two sentences from English of varying degrees of syntactic elaboration. The example in (2) from English illustrates a typical transitive TP (Tense Phrase), where each argument is generated in its own layer: the agent (Maria) in the vP layer, and the patient (corn) in the VP/SC layer. Being the highest noun phrase in the structure, the agent moves to the TP layer to become the sentence subject. While the TP in (2) is a highly hierarchical structure, its bottom layer (VP/SC) is not hierarchical. Somewhere between the two extremes, one can construct a hierarchically less complex sentence in (3), which lacks the agent and the vP layer, and in which it is the patient (corn) that moves to TP to become the subject.

This hierarchy, widely adopted in theoretical syntax, allows a precise reconstruction of the initial stages of syntax. By peeling off the syntactic layers postulated for the modern sentence down to the lowest layer, one arrives at the most basic clausal construct, the universal minimal structure—a VP/SC (Small Clause). The novelty of this proposal is that it isolates an *intransitive absolutive-like* two-slot grammar as a plausible precursor for all syntactic elaboration in the evolution of human language (and language acquisition)^4^. The absolutive-like stage exhibits an underspecified type of grammar capable of accommodating only a single argument, without grammatical means to distinguish subjects from objects. This is reminiscent of the modern absolutive case (contrasting with ergative case), (in) famous for blurring the distinction between subject-like and object-like arguments, as discussed in more detail below. Progovac (2016a) has proposed that this intransitive one-argument foundation is the common denominator for attested language variation in the expression of transitivity/argument structure, including ergative absolutive, nominative-accusative, and serial verb patterns (see also Mithun, 1991, for additional but much rarer possibilities).

In other words, the SC/VP provides the common core that languages share, and a starting point for syntactic elaboration, including for the various ways transitivity is expressed cross-linguistically. By itself simple and straightforward, this foundational structure can be tinkered with by enhancing it and/or reduplicating it in a variety of ways, leaving it up to the creativity of cultures to converge on a solution for transitivity. This is in the spirit of Mufwene's (2013) proposal that language variation in general boils down to different solutions to similar problems. This reasoning dates back to now famous evolutionary scholar, François Jacob, and his claim (1977) that different tinkerers likely develop different solutions to similar problems, such as different types of eyes. Natural selection tends to use materials at its disposal to form a variety of adaptations to similar challenges. Language variation in transitivity, we contend, can be understood in the same light. With rare exceptions, transitive structures typically add only one extra argument. In case of ergative-absolutive languages, the second, ergative-marked argument is “higher” than the first argument, while in nominative-accusative languages, the second, accusative-marked argument is “lower” than the first argument. In languages that have serial verb constructions, one finds multiple intransitive (small) clauses strung together, often just two. It is noteworthy that the enhancements of the foundational pattern are neither radical nor unrestricted, but rather modest and circumscribed. In this respect, they are consistent with typical recombination and replication processes that characterize evolution more generally.

Thus, this proposal provides a way to deconstruct the emergence of transitivity into specific small steps, consistent with typological variation, which in turn reveal communicative pressures and benefits for evolving transitivity. Importantly, we do not propose that the evolution of syntax is a sequence of discrete stages, where each stage limits one to only certain, endemic to that stage, grammatical structures. It is rather a patchwork quilt of partially overlapping, coexisting grammars, accrued throughout language evolution. We propose that the relatively uniform and modest beginnings for grammar, supported by more ancient and more diffuse neurocognitive processing strategies, were followed by gradual enhancements and enrichments of both the grammars and the neural networks that support them. We test this hypothesis in an fMRI experiment reported below.

It is fair to point out that gradualist approaches to evolution have been argued against in biology with a reference to the idea of punctuated equilibrium by Gould and Eldredge (Eldredge and Gould, 1972; Gould and Eldredge, 1977) which was interpreted by some as a refutation of the gradualist, incremental evolution of complex adaptation. However, contrary to this interpretation, Gould and Eldredge did not refute Darwinian adaptationism, nor proposed instantaneous evolutionary change. As discussed at length in Dawkins (1996) and Fitch (2010, pp. 46–53), situating Darwin's writings within the context of the debates of his own time, one can clearly see that Darwin was not a constant-rate gradualist, as is sometimes alleged by punctuated equilibrium advocates, but that his proposal merely allowed incremental changes. As Fitch (2010, pp. 48–49) explained, “gradual speciation and discrete mutation are not in conflict, but offer explanations at a different level of analysis,” the former at the level of the population, and the latter at the level the individual. Thus, we maintain that there are no obstacles coming from biology for studying language evolution as proceeding in small, incremental steps. This approach to the evolution of language is consistent with the many-genes-of-small-effects approach, pursued more recently by Fisher (2017); cf. also Dediu (2015) and Dediu and Ladd (2007). We also contend that there are no such obstacles coming from linguistics either, even though, understandably, there are opposing views^5^. The syntactic analysis we adopt as our starting point here is also in sync with a recent development in the field of linguistic typology. Interestingly, typological research has found it useful to steer away from formulating implicational universals based on transitive structures, such as SOV, SVO, VSO, etc., as originally formulated in Greenberg (1966). Instead, more precise and more insightful universals can be formulated by breaking down transitivity into binary parameters, SV/VS and OV/VO, as they are better predictors of various correlations (Dryer, 1992, 1997; Croft, 2003). This is another argument in favor of decomposing transitivity into smaller structures, with verb and only one argument at a time. We believe that our approach brings together insights and analyses from a variety of different frameworks and approaches to language, each illuminating its different facets.

### The co-existence of “linguistic fossils” with more modern syntax

Jackendoff (1999, 2002) introduced the influential idea of linguistic fossils, i.e., structures occurring in present-day languages, hypothesized to approximate ancient language. Jackendoff considers compounds (e.g., *snowman, scarecrow*), as well as other “loose” combinations, such as adjunction processes (e.g., the attachment of adjectives and adverbs), as good candidates for syntactic fossils. Progovac (2015a, 2016a) builds on this idea to argue that intransitive absolutive-like structures are available in various guises as “living fossils” in modern languages, and that these structures also serve as the foundation upon which a modern sentence gets constructed. The relevant fossils include, but are not limited to, sentences with one absolutive argument in an ergative language such as Tongan (4), as well as the so-called “middles” in an accusative language such as Serbian (6). The fossils in (4, 6) are argued *not* to project the specialized transitivity layer (such as a vP), rendering them “flatter” than their transitive counterparts, in the sense that they project fewer layers of syntactic structure. In other words, the structures of (4, 6) are comparable to the structure in (3) in Figure 1, lacking a vP, and not to the structure in (2) in Figure 1, which projects a vP. Moreover, the only argument in (4, 6) is not thematically specified as either agent or patient, often resulting in vagueness, as indicated by multiple translations, and as captured semantically in (5, 7).

(4)   Oku   ui   ‘a   Mele.    (Tchekhoff, 1973, p. 283).

       pres   call  abs  Mary

      ‘Mary calls.'/ ‘Mary is called.'

(5)  ∃ e [C(e) ∧ Participant (Mary,e)]

       [i.e.: There is an event e such that e is Calling, and

       Mary is a Participant in e.]

(6)   Deca   se   tuku.

       childrense   hit

      ‘Children hit/are hitting each other.'

      ‘Children hit/are hitting themselves.'

      ‘Children hit/are hitting somebody (else)/me/us.'

      ‘One hits/is hitting children.'

(7)   ∃e [H(e) ∧ Participant (Children, e)]

        [i.e: There is an event e such that e is Hitting, and

        Children is the Participant in e.]

Gil (2005) has made a comparable argument for certain one-argument sentences in Riau Indonesian, where there is no case marking (8). He has proposed that their interpretation is simply achieved by an association operator, which associates the meanings of the two words/concepts^6^.

(8)   Ayam       makan

       chicken     eat

      ‘The chicken is eating.'

      ‘Somebody is eating the chicken.'

What is surprising about these one-argument sentences/clauses is that there is no structural/grammatical differentiation between the agent (subject) and the patient (object). As pointed out in Tchekhoff (1973) for Tongan, it would be erroneous to analyze the distinct interpretations in (4) as involving two distinct syntactic structures, one in which Mary is the agent/subject, and the other in which Mary is the patient/object. Absolutive agents and absolutive patients in Tongan are clearly unified grammatically into a single, absolutive role. Instead, Tchekhoff (1973, p. 283) characterizes the pattern in (4) as vague/unspecified, where *Mary* is neither an agent nor a patient, and the two available translations in (4) just reflect a nominative-accusative bias. In his own words, in (4) “Mary is the only determiner [i.e., argument, L.P.], and the whole utterance gives us only the following information: present tense, verb *call, Mary*. And we don't know whether Mary was the agent of the calling, or the recipient of it. Nothing in the Tongan original informs us as to this particular point.” If so, then (4) should be analyzed semantically as in (5), where *Mary* is just an underspecified proto-participant role in the event of calling. Dowty (1991) has advanced the idea of proto-roles for proto-agents and proto-patients. The idea is that proto-agents range over a variety of roles along the agent (volitional) continuum, and that proto-patients range over a variety of roles on the patient (affected) continuum, with the boundary between the two macro roles not rigidly demarcated. The data and analysis presented above lead to conclusion that language can operate with an even more general proto-role: proto-participant role.

Similar considerations hold of Serbian middles, as per the discussion and analysis in Progovac (2015a; 2015b). These *se* constructions involving just one argument are thematically unspecified, and (6) can be interpreted in a variety of ways that involve an event of hitting, and children as participants in that event, as semantically captured in (7). Which interpretation will be salient depends on the context and the pragmatic plausibility, but is not determined by the grammar (see Marelj 2004, who also discusses some aspectual restrictions on the interpretation of middles). The only expressed argument can be either agent-like or patient-like, or even both at the same time, in the case of the reflexive interpretation.

Importantly, Serbian (6) sharply contrasts with the transitive accusative counterpart in (9), which has only one interpretation, and which is standardly analyzed as projecting the transitivity (vP) layer. In this respect, the example in (9) corresponds to the structure in (2) in Figure 1, rather than to the structure in (3) in Figure 1, which lacks the vP.

(9)       Deca    me/ga      tuku.

           children me/him hit

          ‘The children are hitting me/him.'

(10)    ∃e [H(e) ∧ Participant (Children,e) ∧ Patient

           (Me/Him,e)]

           [i.e., There is an event e such that e is Hitting, and

           Children is the Participant in e, and Me/Him is the

           patient in e.]

Here the accusative argument (me/him) is added from below, to instantiate a patient/object, which then renders the other participant, the unmarked (nominative) argument, as a thematically higher argument, in this case agent/subject. This analysis allows the proto-layer to retain its underspecified character, with one proto-participant argument, given that the specification of the second, added argument is sufficient to disambiguate.

A comparable (but opposite) situation holds in Tongan, where an extra (ergative) argument is a higher argument (in this case agent), added on the top, while the other participant, the unmarked (absolutive) argument, is rendered as a thematically lower argument (in this case patient). Again, the analysis allows for the proto-layer to be preserved as essentially intact, with one proto-participant argument, given that the added (ergative) argument is sufficient for disambiguation.

(11)    Oku       ui       ‘e           Sione   ‘a         Mele

          pres     call     erg     John     abs     Mary

          ‘John calls Mary.'

(12)    ∃e [C(e) ∧ Agent (John,e) ∧ Participant (Mary,e)]

           [i.e.,: There is an event e such that e is Calling, and

           John is the Agent in e, and Mary is a Participant

           in e.]

It is significant that both patterns (ergative and accusative) have as their foundation an intransitive small clause with one verb and one unmarked argument, a proto-participant in the event, which reflects the initial stage of syntax as reconstructed in Progovac (2015a, 2016a)^7^.

This evolutionary characterization of ergative and accusative cases as secondary, tacked-on cases, is well aligned with the Dependent Case Theory explored in Yip et al. (1987), and Marantz (1991), the approach that has recently been revived in the work of McFadden (2004) and Baker (2015), among others. According to Marantz (1991, p. 24), “dependent case is what we will call accusative and ergative… Accusative is the name for the dependent case that is assigned downward to an NP position…Ergative is the name for the dependent case assigned upward to the subject position….” This same insight was adopted in Baker (2015, pp. 48–49). In other words, accusative and ergative are cases dependent on the presence of another (first) argument. The evolutionary approach explored here can shed light on various otherwise unexplained phenomena, including on the small clause foundation for all sentences, and on the nature of language variation in the expression of transitivity.

The following question arises in this respect: why would this small clause foundation be there at all, and why would it *still* be there even today, after many millennia of evolution? There are two reasons. First, evolution of complex adaptations typically takes place gradually, in incremental small steps, which would explain why the small clause step was useful, or even necessary, in the evolution of human language. Gradual evolution is often described as a “tinkering” process, where new, slightly more complex elements are only created by tinkering with the existing elements. In his highly influential and insightful essay, Jacob (1977) maintains that natural selection's creative force lies in its ability to combine and recombine old material into novelties, in a process that resembles imperfect tinkering, rather than engineering (see also Fitch, 2010, p. 55, who reinforces this view). Crucially, as Jacob points out, evolution hardly ever produces complex traits from scratch (see also Pinker and Bloom, 1990), which is why the small clause one-argument beginnings may have provided necessary scaffolding for the assembly of more complex syntactic patterns. Second, if the basic small clause was a useful and robust stage in the evolution of human language, then rather than being discarded, it has persisted as a necessary foundation/scaffolding that facilitates the elaboration/tinkering of additional structure, including in language acquisition.

### Neuroscientific hypotheses: transitivity and the basal ganglia in an fMRI experiment

The coexistence of proto-syntactic fossils with more modern syntactic structures in present-day languages provides a fertile ground for testing evolutionary hypotheses using neuroimaging. Our study focused on the Serbian middles contrasted with canonical transitive structures; both are highly productive in Serbian. As discussed above, in contrast to the flatter middles, the transitives in Serbian are analyzed as projecting an additional layer of structure, an active vP layer, which renders them more hierarchical. We hypothesized that Serbian middles, relative to matched transitive accusative structures, will result in reduced activation in regions associated with syntactic processing, specifically in the Broca's–Basal Ganglia network. Our main hypothesis ties directly into the findings that the connectivity of the Broca's–Basal Ganglia network was bolstered relatively recently in evolution, in the line of descent of humans (see e.g., Enard et al., 2009; Hillert, 2014; Dediu, 2015; for the role of FOXP2 and other genes in this respect). We also hypothesized possible increased activation in regions not typically associated with syntactic processing when examining the reverse, that is, the activation in middles relative to full transitive structures. This would be consistent with Bisang (2009) and Ansaldo et al. (2015)'s claims that syntactically simpler structures may activate more semantic processing.

The basic assumptions behind these predictions are the following. On the one hand, complex grammatical patterns, which arguably only human brains can process, are supported by the most recently evolved/enhanced neural networks. To put it differently, complex hierarchical structures require more support from these recently evolved networks than their flatter proto-syntactic counterparts. On the other hand, the vague/underspecified structures of proto-syntax, which need less support from these most recently evolved networks, may show a more diffuse and less streamlined distribution across the brain, as well as possibly more individual variability.

Whereas middles in Serbian show ambiguity/underspecification with respect to the thematic role of their only expressed argument (cf. e.g., *Deca se tuku* “Children _SE_ hit”) and canonical transitives are unambiguous in this respect (cf. *Deca me tuku* “Children me hit”), it is possible to skew the semantic interpretation toward the “me” readings in middles by selecting exclamatives (13, 14) or imperatives (15, 16), rendering them as close semantic and phonological matches to transitives, differing primarily in the level of syntactic layering/elaboration. The examples in (13, 15) are middles, and the examples in (14, 16) are the corresponding transitives.

(13)    a.    *Mama*,   *Milan*        ***se***     *udara*!

                 Mom,     Milan         se     hits

                ‘Mom, Milan is hitting (me)!

          b.    *Mama*,   *ovaj*          *pas*   ***se***            *ujeda*!

                 Mom,     this           dog    se            bites!

               ‘Mom, this dog is biting (me)!'

(14)    a.    *Mama*,   *Milan*       ***me***     *udara*!

                 Mom,     Milan        me     hits

                ‘Mom, Milan is hitting me!'

          b.    *Mama*,   *ovaj*         *pas*    ***me***           *ujeda*!

                 Mom,     this          dog    me            bites!

               ‘Mom, this dog is biting me!'

(15)    a.    *Ne*       *udaraj*      ***se**!*

                 not       hit.imp       se

               ‘Don't hit (me)!

          b.    *Ne*        *guraj*        ***se***!

                 not       push.imp   se

               ‘Don't push (me)!'

(16)    a.    *Ne*       *udaraj*      ***me**!*

                 not     hit.imp        me

               ‘Don't hit me!'

         b.     *Ne*      *guraj*         ***me***!

                 not      push.imp    me

               ‘Don't push me!'

## Method

### Participants

We tested 14 healthy adults, native Serbo-Croatian speakers (6 Female, age range 21–62, Mean = 33.36, *SD* = 12.23) residing in the US. Participants' self-reported native fluency in Serbo-Croatian was verified by the first author, a Serbian native speaker, who conducted both oral and written interviews with each participant. All the participants were also fluent in English, with 11 participants out of 14 clearly exhibiting non-native command of English^8^. Participants were screened prior to the study and reported no language impairments and no abnormal psychiatric or neurological history. All participants were determined to be right-handed by the Edinburgh Handedness Inventory (Oldfield, 1971). Written informed consent was obtained from all participants, as required by Wayne State University IRB.

### Stimuli and procedure

Our main hypotheses concerned neural correlates of processing middles compared to transitives in Serbian. Each of the two conditions (Middles and Transitives) consisted of two types of sentences: two types of middles, exclamative (see 13 above) and imperative (see 15 above), as constructions most semantically matched to their transitive counterparts, and two types of transitives, also exclamative (see 14 above) and imperative (see 16 above), with a total of 64 unique stimuli used across the two conditions (see Appendix for complete list of stimuli used in this study). Stimuli were presented visually as centered white text on a black background (Times New Roman, 80-point font). Each stimulus was presented for 1,500 ms followed by a 250 ms fixation cross. Stimuli were presented in a block-design, with each block consisting of 8 unique stimuli. There were 4 blocks per each of the 2 conditions of interest. To ensure that participants adequately engaged with the stimuli, we embedded a simple repetition detection task (1-back) in each block, so that one of the stimuli was presented twice in succession and participants were asked to indicate with a button press when such repetition occurred. Each block lasted a total of 15.75 s, and was followed by 10 s inter-block-interval, during which a fixation crosshair was presented in the middle of the screen. Additional conditions were included in the imaging design but are not the focus of this study. In these other conditions, 144 other unique stimuli were presented in 18 blocks with stimuli consisting of either small clauses, full sentences, simple compounds, or complex compounds. The order of blocks presentation was pseudorandomized to minimize condition order effects. Two pseudorandom orders were created and assigned to participants based on whether they had an even or an odd numbered study ID.

Presentation of the stimuli and recording of responses was conducted with PsychToolbox in MATLAB. Accuracy and reaction times were collected and averages were calculated per condition and across participants. Due to technical difficulties accuracy and reaction times could not be calculated for 3 participants; these participants provided complete fMRI data sets and therefore data from these participants were included in all neuroimaging analyses. In addition, data form one other participant was excluded due to noncompliance, as it was evident the participant was sleeping during the scanning session.

### MRI data acquisition and analyses

MRI data were acquired in a 3T Siemens Verio scanner at the Wayne State University MR Research Facility located in Harper University Hospital in Detroit, MI. T1-weighted whole-brain structural images were acquired using an MP-RAGE sequence (176 coronal slices, repetition time (TR) = 1,680 ms, echo time (TE) = 3.51 ms, flip angle = 9°, field of view = 256 mm, 176 × 256 voxels, and voxel size = 0.7 mm × 0.7 mm × 1.3 mm). Functional images during the experimental task were acquired in a single run using a T2^*^-weighted multiband accelerated EPI pulse sequence: 75 slices parallel to the AC-PC plane (TR = 2,000 ms, TE = 30 ms, flip angle = 90° voxel size = 2 × 2 × 2 mm^3^, multiband factor = 3, duration = 11 min and 18 s, total 339 volumes).

Functional data were analyzed using SPM12 package (Wellcome Department of Imaging Neuroscience, London, UK). Images were motion corrected, normalized to the Montreal Neurological Institute (MNI) template, and smoothed with a 6-mm full-width half-maximum Gaussian kernel. Statistical analyses of fMRI data were conducted using general linear modeling (GLM), as implemented in SPM12. First-level analyses included conditions of interest: Middles, Transitives and other conditions not of interest in this study (Small Clauses, Full Sentences, Simple Compounds, Complex Compounds). All conditions were modeled with separate regressors. The BOLD response was modeled by convolving a canonical hemodynamic response (HDR) function with a boxcar function spanning the duration of the block (15.75 s) and temporal derivatives of each block were included in the GLM to account for temporal shifts in the response of the stimuli (Friston et al., 1998).

We assessed activation associated with the processing of Middles compared to Transitives in *a priori* anatomically-defined regions of interest (ROIs) that are part of the known specialized language network involved in syntax-related processing (i.e., Broca's area and the basal ganglia). Four anatomically defined ROIs were generated using the Wake Forest University Pickatlas tool (TD Brodmann areas) spanning the left and right inferior frontal cortex regions identified as Brodmann Area (BA) 44 and 45, and the left and right basal ganglia (combined caudate and putamen). These ROIs were used to extract average activation per condition. Planned comparisons were conducted with the mean extracted contrast values from each of the four ROIs described above (all reported findings from these analyses were significant at *p* < 0.05). We tested significant effects using a paired *t*-test (2-tailed), Bonferonni-corrected for multiple comparisons.

In addition, with group-level whole-brain analyses we set to identify the regions in which there was an increase in activation for Transitives compared to Middles, and to examine whether regions evinced the opposite trend, an increase associated with the processing of flatter structures, which may rely on more general cognitive processing strategies available prior to the emergence of complex human language, and the concomitant modification of the human brain. Thus, the contrasts of interest—Middles versus Transitives—were computed on the individual level and combined in a whole brain group-level analysis. Group-level activation maps were threshold at *p* < 0.001 at the voxel level, with an extent threshold of 100 contiguous voxels for the cluster level, minimizing the possibility of the findings being false positives.

## Results

### Behavior

Overall, participants were highly accurate in responding to repeated stimuli (mean ± SD: 0.98 ± 0.03), suggesting that participants were engaged and processing the presented stimuli. Accuracy rate in this detection task did not differ between Middles (1.00 ± 0.00) and Transitives (0.98 ± 0.08), *t*_(9)_ = 1.00, *p* = 0.34. Mean reaction time in the detection task was slightly longer [*t*_(9)_ = 2.43, *p* = 04] in the Middles (640 ± 160 ms) compared to the Transitives condition (590 ± 140 ms), suggesting the possibility of increased demands for processing Middles compared to Transitives.

### Reduced brain activation for middles compared to transitives: syntax-related rois

**Figure 2:**
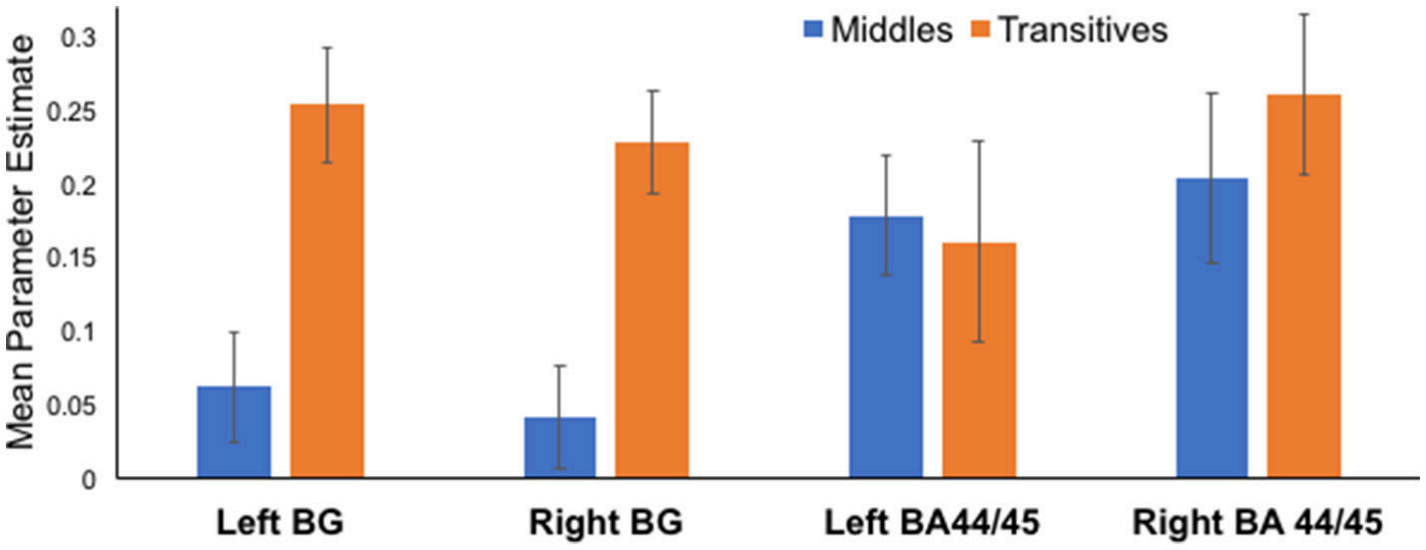
Differential activation for Middles compared to Transitives in basal ganglia and BA 44/45 ROIs. In both left and right basal ganglia ROIs there was marked increase in activation for Transitive compared to Middles. Activation in left and right BA44/45 ROIs was associated with the processing of both types of stimuli.

Parameter estimates from *a priori* anatomically identified regions known to be involved in the processing of syntax were used in the main analyses. Specifically, we extracted parameter estimates for Middles and Transitives for 4 ROIs: bilateral BA 44/45, and bilateral basal ganglia. Extracted values per each condition are presented in Figure 2 to allow comparisons across conditions. Statistical tests were only conducted in predefined comparisons using *t*-test in bilateral basal ganglia, and bilateral BA 44/45.

Processing of Transitives, compared to Middles, was associated with an increase in activation in the basal ganglia ROIs both on the left [Transitives: 0.25 ± 0.14, Middles: 0.06 ± 0.13, *t*_(12)_ = 5.64, *p* < 0.001] and the right [Transitive: 0.23 ± 0.12, Middles: 0.04 ± 0.12, *t*_(12)_ = 4.57, *p* = 0.001]. In contrast, in BA 44/45 ROIs activation was evidenced for both Transitives and Middles with no significant differences between conditions in the left [Transitives: 0.18 ± 0.20, Middles: 0.18 ± 0.14, *t*_(12)_ = 0.25, *p* = 0.83], and a non-significant trend in the right [Transitives: 0.20 ± 0.17, Middles: 0.13 ± 0.18, *t*_(12)_ = 1.87, *p* = 0.09 uncorrected].

### Reduced brain activation for middles compared to transitives: whole-brain analyses

**Figure 3:**
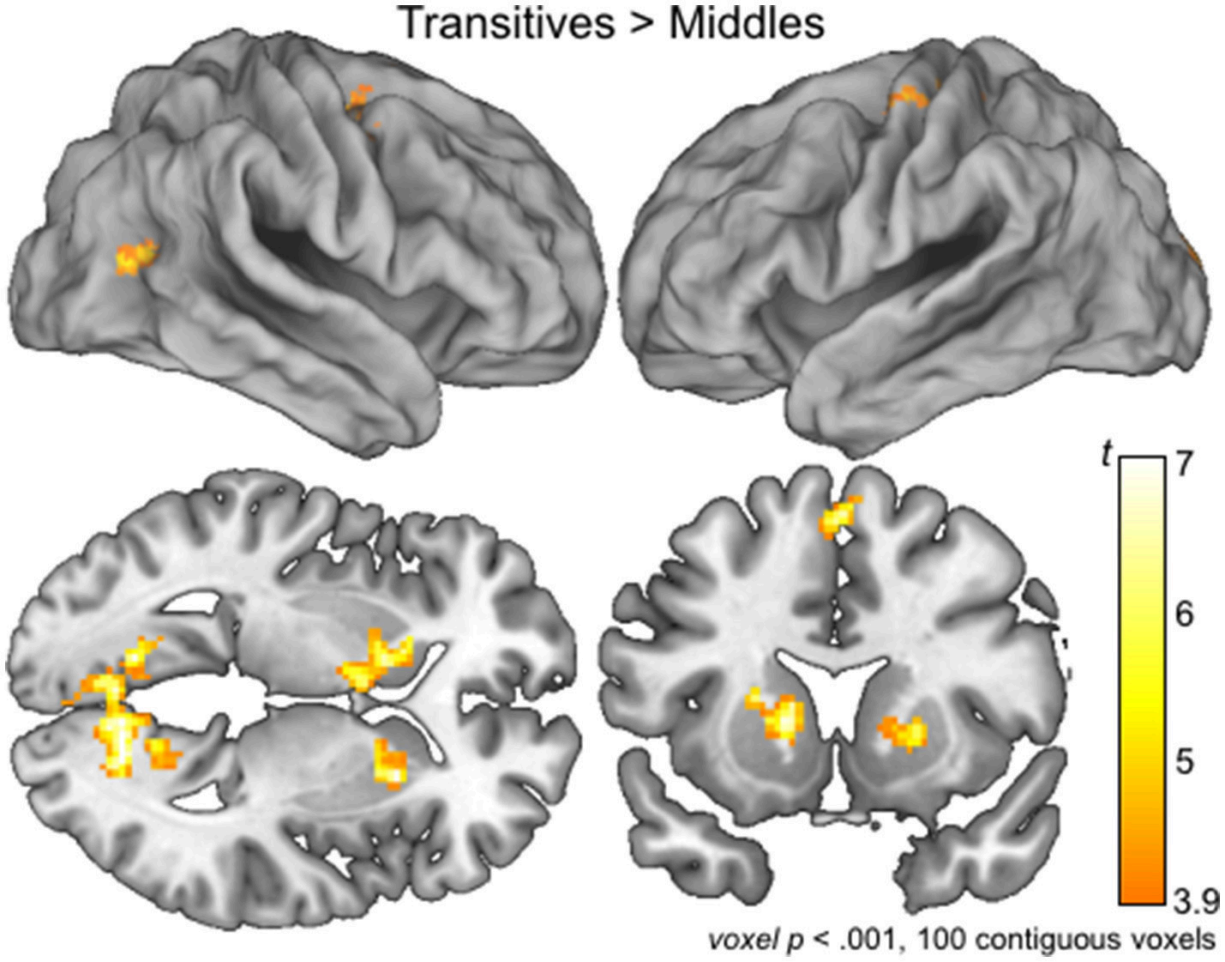
Brain activations associated with the processing of Serbian Transitives compared to Middles. Group level whole brain activations maps for the contrast Transitive > Middles are overlaid on lateral view of the right (top, left) and left (top, right) hemispheres, and on a sagittal (bottom, left) and coronal (bottom, right) slices at the level of the basal ganglia. See Table 1 for detailed list of activations.

To confirm the involvement of language related regions in the processing of Transitives compared to Middles, we used a planned exploratory analysis conducted at the whole-brain level by created group-level model of the first-level, individual subject contrasts of activation associated with the processing of Transitives compared to Middles. Brain regions in bilateral basal ganglia, as well as regions in the precentral gyrus (BA 6) and visual cortex including bilateral lingual gyrus (BA 30) showed more activation for Transitives compared to Middles (Table 1; Figure 3). BA 6 plays a role in planning coordinated movements, and has also recently been linked to language related processing (Kambara et al., 2017; Nishida et al., 2017). Indeed, according to Ardila et al. (2016b), “‘Broca's complex’ includes not only left BA44 and BA45, but also BA46, BA47, partially BA6 (mainly its mesial supplementary motor area) and extending subcortically toward the basal ganglia and the thalamus.”

**Table 1:** Brain activations associated with processing Serbian Transitives compared to Middles.

**Hemisphere**	**Region**	**BA**	**MNI coordinates**			***T* Values**	**Number of voxels**
			**x**	**y**	**z**		
**A. TRANITIVES** > **MIDDLES**							
Right	Putamen	NA	24	16	2	7.33	103
	Caudate		12	10	4	5.25	
Left	Caudate	NA	−6	6	4	7.06	281
	Putamen		−18	10	4	6.82	
Right	Precentral gyrus	6	36	−6	42	11.20	308
Left	Precentral gyrus	6	−30	−4	62	6.84	383
Left	Medial frontal gyrus	6	0	10	60	8.89	459
Right	Middle temporal gyrus	39/19	42	−60	10	7.97	132
Left/Right	Lingual syrus	30	−4	−72	4	10.61	1511
Right	Cerebellum	NA	42	−52	−30	7.51	139
**B. MIDDLES** > **TRANSITIVES**							
No voxels survived the threshold							

*p < 0.001, 100 contiguous voxels*.

Whole-brain group-level analysis allowed us to assess the possibility of unique regions supporting the processing of Middles when compared to Transitive syntactic structures. To test this possibility, we used the contrast to identify increased activations associated with the processing of Middles compared to Transitives. This contrast did not identify any brain regions in which activation was increased for Middles compared to Transitives. These findings are consistent with a possibility that processing of middles relies on strategies that may be different in different individuals and therefore no consistent findings can be identified at the group level.

## Discussion

We found support for our main hypothesis that processing of less hierarchical structures would be related to reduced activation in the brain networks associated with syntactic processing, namely in basal ganglia. Although we did not find an effect in Broca's area, transitives, compared to middles, evoked greater activation in the precentral gyrus (BA 6), proposed to be part of the “Broca's complex” (e.g., Ardila et al., 2016b). Our finding of the role of basal ganglia is consistent with a number of previous studies, which found that Broca's area is not the sole center for syntactic processing, but rather is part of a larger circuit that involves subcortical structures (Gibson, 1996; Lieberman, 2000, 2009; Vargha-Khadem et al., 2005; Ullman, 2006; Ardila et al., 2016a,b). While in this study we did not find differential activation in the Broca's region, in a related study we found greater activation in both Broca's region and basal ganglia in the more complex syntax condition (Progovac et al., in preparation). There we investigated the processing of English small clauses (SCs) (e.g., *Point taken; Problem solved*) relative to their tensed sentential counterparts (TPs) (e.g., *The point is taken; The problem is solved*). Increased activation was found in the left BA 44 and in the right basal ganglia for more hierarchical tensed clauses relative to the flatter small clauses, even after controlling for the sentence length effect, indicating that greater activation in these regions was not merely due to differences in the length of word strings, but rather was likely related to the presence of extra layers of syntactic structure^9^.

The key difference between that study and the experiment reported in the current article was that the latter tested the impact of the vP (transitivity) layer, while the experiment with English clauses tested the impact of the TP layer, as well as the DP layer, which is required by the TP in English. In other words, unlike the small clauses in (17), the TP sentences require their subjects to be DPs, as indicated in the contrast between (18) and (19) below (see Progovac, 2013 and references cited there for the details of the analysis of these clauses).

(17) Problem solved. Point taken. Mission accomplished.
(18) The problem is solved. The point is taken. The mission is accomplished.
(19) ^*^Problem is solved. ^*^Point is taken. ^*^Mission is accomplished.

We offer the following tentative suggestions for the difference in the findings between the two experiments regarding the Broca's area. While the two findings overlap in their isolation of the involvement of basal ganglia, only the TP/DP condition has also identified an effect in the Broca's area. One possible explanation may be that even though the vP layer is hierarchical, it is more basic and less abstract than the TP or DP layers, as it still deals with content words, nouns and verbs. The TP and DP layers involve abstract, purely grammatical categories, such as tense auxiliaries (*is, was*) and articles (*a, the*). While transitivity can be seen as instantiating hierarchy, but not a high level of functional abstraction, the TP and DP layers instantiate both hierarchy and a high level of functional abstraction, potentially resulting in higher processing demands. Thus, it is plausible that the transitivity (vP) layer, being a more basic (lower) layer of structure and less functional/less abstract in nature than TP/DP layers, is less reliant on Broca's region and/or requires less dense connectivity between the Broca's region and basal ganglia.

Another possible explanation for the different findings may be the degree to which the constructions that we examined in the two experiments differed from each other. Namely, while the Serbian vP condition (transitives vs. middles) differed only in one layer of structure (the vP layer), the TP/DP condition in English differed in two layers of structure (TP and DP). Perhaps the layering of functional projections has a cumulative effect on processing, with more such layers requiring more neuronal interconnectivity (see also Footnote 10). Future experiments can be designed to tease these options apart, and we suspect that a variety of languages, as well as a variety of constructions, will need to be tested before a clear picture emerges.

Our finding of the differential involvement of basal ganglia in the processing of more complex syntax is especially significant in light of the recent studies that have identified the BA 44–Basal Ganglia network as a syntactic processing network with very strong neural interconnectivity (Ardila et al., 2016a). The basal ganglia are a collection of subcortical structures on both sides of the thalamus, outside and above the limbic system, below the cingulate gyrus and within the temporal lobes. The largest group of these structures is the striatum, consisting largely of the caudate nucleus and the putamen. The basal ganglia are highly interconnected to cortical regions, especially in the frontal lobes, including Broca's, via parallel anatomically and functionally segregated “loops” (Draganski et al., 2008; Frey et al., 2008; Ford et al., 2013). The involvement of the striatum in syntactic processing was demonstrated across different languages in lesion studies, including in patients with Parkinson's and Huntington's disease (Moro et al., 2001; Teichmann et al., 2005, 2008; Newman et al., 2010). Furthermore, studies produced experimental evidence for a language-basal-ganglia-gene pathway using animal models. For example, in one study (Enard et al., 2009), two amino acid substitutions, thought to have been positively selected for in the course of human evolution because of their enhancing effect on language, were introduced in the *FOXP2* gene of mice. These genetic mutations did not result in alterations in the general health of mice, but triggered changes in their ultrasonic vocalizations, exploratory behavior and decreased dopamine concentrations in the brain. Most relevant for our study, the humanized *FOXP2* alleles affected the basal ganglia, increasing dendrite lengths and synaptic plasticity of the medium spiny neurons in the striatum.

Our study offers some encouraging preliminary evidence, in need of further confirmation, for the proposal that relatively uniform and modest beginnings for human language (i.e., proto-syntax prior to the emergence of more hierarchical grammar), supported by more ancient brain processing strategies, were followed by a (gradual) enrichment of the grammar, accompanied by more streamlined processing strategies and more densely interconnected neural networks needed to support them (see also Ansaldo et al., 2015). Our results and the approach we explore here are compatible with the idea that recent genetic mutations, including in *FOXP2*, in the line of descent of humans, increased synaptic plasticity and neuronal connectivity of the human brain (e.g., Hillert, 2014; Dediu, 2015), particularly in the frontal-striatal network, enabling human capacity for more complex language. This is where our proposal addresses the language-brain-gene connections.

The involvement of *FOXP2* in language and frontal-striatal brain network was directly documented by a discovery that showed that individuals heterozygous for *FOXP2* alleles with a certain mutation affecting the forkhead DNA binding domain of the protein suffered from a developmental impairment affecting speech and language (Lai et al., 2001). In the widely publicized case of the KE family, whose congenital language impairment was caused by a mutation in the *FOXP2* gene (Fisher et al., 1998), language deficits included impaired understanding of complex syntax and grammatical morphology (Gopnik, 1990) and difficulties with articulation, among several other symptoms. The morpho-syntactic deficits included subject drop and the nonsystematic use of plural forms and tense (e.g., Gopnik and Crago, 1991; see also Piattelli-Palmarini and Uriagereka, 2011). This implicates problems with higher functional categories, including tense and TP. Liégeois et al. (2003) showed that the affected KE members not only exhibited under-activation in the Broca's area and its right homolog, but that both the caudate nucleus and putamen were sites of morphological abnormality in the affected members. Impairments in the members of the KE family were not restricted to language. In addition, the affected family members had symptoms of verbal apraxia and lowered nonverbal cognition (Vargha-Khadem et al., 1995), suggesting complex wide-ranging effects of the *FOXP2* gene.

Recent comparative work that examined linguistic phenotypes across developmental disorders affecting language acquisition converged on the observation that narrow syntax (i.e., the internal computational system) tends to be more resilient than usually thought, i.e., likely to be preserved in many forms of developmental pathology, which by and large affect linguistic phenomena related to externalization, i.e., morphosyntax and morphophonology, linguistic domains that interface with the Intentional-Conceptual and Sensory-Motor systems (The Locus Preservation hypothesis; Leivada et al., 2017; Lorenzo and Vares, 2017)^10^. We would argue that dissociation in the patterns of language breakdown may reflect distinct underlying brain network organization corresponding to distinct evolutionary stages during which these linguistic domains emerged.

Further investigations in the function of *FOXP2* and developmental language disorders revealed that *FOXP2*, a regulator gene, is not the “grammar” or even the “language gene”, given that (i) it is expressed not only in multiple areas of the brain, but also in other organs, serving a variety of functions, and, that (ii) most common cases of developmental language disorders that entail grammatical impairment do not involve mutations in *FOXP2* (Rakhlin and Grigorenko, 2015). However, it is clearly part of the genetic network relevant to language. Since the discovery of *FOXP2*, it has become increasingly clearer that genetic bases of language are complex and involve many genes of small effect, including genes that, like *FOXP2*, act pleiotropically (i.e., serve multiple functions). The case of the KE family is one of the rare cases of a monogenetic language disorder: the majority of cases of developmental language disorders do not involve a major single causal gene, but rather a constellation of genes, each exerting only a modest effect (raising the risk of a language disorder in an individual by a small percent) and interacting with environmental effects in a complex way. Notably, one of the implicated genes is a gene down-regulated by *FOXP2, CNTNAP2*. This gene has not only been linked to language impairment in children (Vernes et al., 2008), but also to normal variability in language development at the age of 2 years (Whitehouse et al., 2011), and to distinct patterns of neural activation on ERP language tasks (Kos et al., 2012) and fMRI (Whalley et al., 2011) in healthy adults.

A gradualist approach to the evolution of syntax we subscribe to is entirely consistent with the multiple-genes-of-small-effect view and does not expect there to be a grammar gene, i.e., a major causal gene of large effect, such that a single mutation in this gene would give rise to complex hierarchical syntax. Instead, a gradualist approach lends itself well to the modern understanding that pathways connecting genes to complex phenotypes, such as language, are complex and nonlinear, as articulated in Dediu (2015, p. 10944) and Dediu and Ladd (2007). While the outline of the puzzle is becoming increasingly clearer, more studies combining insights from theoretical linguistics, neuroscience, and developmental language disorders will be essential for advancing this line of research. The case of KE family is exceptional but consistent with this view: a single mutation in the gene caused severe disturbances in language processing and language use, but not complete loss of language), as well as disturbances in functions outside of language. This case also distills the essence of our evolutionary proposal, with all the components of an evolutionary approach to language coming together: a genetic change (a harmful or beneficial mutation), a linguistic change (impairment or development), and a distinct processing path associated with evolutionary development.

## Conclusions

The knowledge generated in the fields of genetics, neurophysiology, developmental cognitive neuroscience, and linguistics is rapidly growing, and it is difficult to predict how our understanding of the evolution of language may shift in light of future discoveries. However, our present knowledge is consistent with the conclusion that genetic mutations in *FOXP2* and other genes played an important role in the enhancement of the frontal-striatal brain network, densely connecting Broca's area to the basal ganglia, which in turn facilitated effortless processing of ever more and more complex syntax. The approach outlined here provides a good model for probing these questions further, and for shedding direct light on both language commonalities and language variation, a model that enables sustained cross-pollinating influences among the fields of linguistics, neuroscience, and genetics.

## Ethics statement

This study was carried out in accordance with the recommendations of Wayne State University IRB Committee guidelines, with written informed consent from all subjects. All subjects gave written informed consent in accordance with the Declaration of Helsinki. The protocol was approved by Wayne State University IRB Committee.

## Author contributions

LP provided theoretical framework and linguistics background. NR provided speech language pathology and genetics background. WA and RL ran the experiments and did statistical calculations. LT provided help with statistical and other analyses. NO provided the fMRI expertise and oversight.

### Conflict of interest statement

The authors declare that the research was conducted in the absence of any commercial or financial relationships that could be construed as a potential conflict of interest.

## Footnotes

^1^There are additional projections that have been postulated for modern sentences in syntactic literature, such as Aspect Phrases (AspPs), Agreement Phrases (AgrPs), Topic Phrases (TopPs), etc. Our focus is on the syntactic layers in (1) because they meet with wide acceptance, and have the widest utility in the syntactic analysis of a variety of languages, as they relate to: transitivity, finiteness, and subordination.

^2^A related claim is found in the title of Dobzhansky's (1973) article “Nothing in biology makes sense except in the light of evolution.” On the other hand, as pointed out by a reviewer, there are several linguists who do not adopt this view, and who argue that language does not evolve but only changes (see e.g., Lightfoot's (in press); article titled “Nothing makes sense except in the light of change”). That language constantly changes is undeniable, and easily observable, but that does not mean that there are no selection forces acting upon language. As stated in Fitch (2008, p. 522), “language change does not entail a cessation of selection” (his article is subtitled “There is no ‘logical problem of language evolution”’). Steels (2011), who advocates studying cultural evolution (language change), also considers that both biological and cultural evolution are necessary to explain language, as these two processes propel each other through a self-reinforcing spiral process. In other words, we do not deny the relevance of language change—on the contrary, we believe that language change is the driving force behind evolution. But we do not subscribe to the view that language change, and language in general, are exempt from evolutionary processes, and we do not see the studies of language change as alternatives to the studies of language evolution, but rather we see them as two different facets of the same phenomenon.

^3^The CP layer is especially relevant for embedded clauses/subordination, or for sentences with *wh*-movement, neither of which are discussed in this article.

^4^Arguably, the intransitive small clause constitutes not only the foundation of syntactic structure elaboration in language evolution, but also in language acquisition. Thus, majority of verbal utterances in the speech of children learning a variety of languages at the onset of syntactic development (early verbal combinations) are analyzable as absolutive-like small clauses, with the additional layers emerging gradually (e.g., Rakhlin and Progovac, 2017).

^5^Many arguments used by saltationist approaches to the evolution of language, such as those in Lightfoot (1991) and Berwick and Chomsky (2011; 2016) have been responded to in e.g., Progovac (2009, 2015a, 2016b).

^6^Gil's association operator allows other possible interpretations for (8), including “the chicken that is eating,” which are not available in the sentences in (4) or (6). This may be due to the presence of specific functional categories in the latter, such as tense/agreement marking, which flag these as finite sentences. In general, the presence of functional categories tends to delimit the range of possible interpretations, leading to more precision/less vagueness.

^7^For completeness, we offer a representative example of the third transitivity type, a serial verb construction from Gungbe (Aboh, 2009), the structure that is not the focus of this paper: (i) Àsíbá     bέ       lέsì      έù    Asiba    collect   rice     eat    ‘Asiba ate a lot of rice.' We observe that in (i) each verb has only one argument, but this seemingly bi-clausal structure results in a single transitive clause interpretation. As Aboh (2009) makes it clear, the verb series in today's languages involves various grammatical complexities, and variation across languages, and this is also the case with ergative-absolutive (e.g., Aldridge, 2008) and nominative-accusative patterns.

^8^We recognize that using bilingual speakers is a limitation of the study and a replication of this experiment would benefit from being conducted with monolingual speakers of Serbo-Croatian.

^9^However, our experiment with Serbian small clauses vs. full TPs did not yield a significant result. While the issue deserves further attention, it is possible that the distinction between a small clause and a sentence is more subtle/more fluid in Serbian, given that it is only marked by the absence vs. presence of an auxiliary verb, whereas in English the presence of the tense auxiliary is necessarily accompanied by the presence of a determiner, as discussed below in the text. This indicates that English requires a DP (Determiner Phrase) projection with full TPs, while Serbian does not (see e.g., Bošković, 2008).

^10^We thank an anonymous reviewer for bringing this literature to our attention.
