# Supplementary material for: Diversity of Grammars and Their Diverging Evolutionary and Processing Paths: Evidence From Functional MRI Study of Serbian
Source: Front Psychol. 2018 Mar 6;9:278. doi: 10.3389/fpsyg.2018.00278 (PMC5845673; doi:10.3389/fpsyg.2018.00278)
Supplement: Supplementary file 1 [file DataSheet1.pdf]

Appendix: Stimuli

A) Exclamative middles

Deca se tuku!
Pas se ujeda!
Oni se prskaju!
Milan se udara!
Petar se gura!
Oni se pljuju!
Petar se gadja!
On se vredja!
Milan se čupa!
On se vuče!
Ona se ujeda!
Milan se grebe!
Ona se češe.
Milan se dira!
Ona se pipa!
On se štipa!

B) Imperative middles

Ne tuci se!
Ne ujedaj se!
Ne prskaj se!
Ne udaraj se!
Ne guraj se!
Ne pljuj se!
Ne gadjaj se!
Ne vredjaj se!
Ne čupaj se!
Ne vuci se!
Ne ujedaj se!
Ne grebi se!
Ne češi se!
Ne diraj se!
Ne pipaj se!
Ne štipaj se!

C) Exclamative Transitives

Deca me tuku!
Pas me ujeda!
Oni me prskaju!
Milan me udara!
Petar me gura!
Oni me pljuju!
Petar me gadja!
On me vredja!

Milan me čupa!
On me vuče!
Ona me ujeda!
Milan me grebe!
Ona me češe!
Milan me dira!
Ona me pipa!
On me štipa!

D) Imperative transitives

Ne tuci me!
Ne ujedaj me!
Ne prskaj me!
Ne udaraj me!
Ne guraj me!
Ne pljuj me!
Ne gadjaj me!
Ne vredjaj me!
Ne čupaj me!
Ne vuci me!
Ne ujedaj me!
Ne grebi me!
Ne češi me!
Ne diraj me!
Ne pipaj me!
Ne štipaj me!
